# Serum Bile Acid Profiling Across the Full Spectrum of HBV-Related Liver Diseases in Chinese Population: Implications for Diagnosis and Treatment Assessment

**DOI:** 10.3390/biomedicines14010084

**Published:** 2025-12-31

**Authors:** Jiahua Mu, Deliang Huang, Lingyun Chen, Guilan Zhu, Guixue Hou, Liang Lin, Jiuxin Qu, Siqi Liu, Jun Chen

**Affiliations:** 1 College of Life Sciences, University of Chinese Academy of Sciences, Beijing 100049, China; mujiahua19@mails.ucas.ac.cn; 2 Department of Proteomics, BGI Genomics, Shenzhen 518083, China; chenlingyun@bgi.com (L.C.); guilanzhu19@gmail.com (G.Z.); houguixue@bgi.com (G.H.); linl@bgi.com (L.L.); 3 Department of Liver Diseases, Shenzhen Third People’s Hospital, The Second Affiliated Hospital of Southern University of Science and Technology, Shenzhen 518112, China; huangdeliang2020@163.com; 4 Department of Clinical Laboratory, National Clinical Research Center for Infectious Diseases, Shenzhen Third People’s Hospital, The Second Affiliated Hospital of Southern University of Science and Technology, Shenzhen 518112, China; qujiuxin@mail.sustech.edu.cn

**Keywords:** antiviral therapy, bile acid profile, biomarkers, chronic hepatitis B, cirrhosis, hepatocellular carcinoma, liver failure, machine learning

## Abstract

**Background/Objectives:** Conventional serum biomarkers such as ALT and AST exhibit limited sensitivity and specificity in distinguishing the spectrum of HBV-related liver diseases, especially chronic hepatitis (CHB), cirrhosis (LC), and hepatocellular carcinoma (HCC). This study aimed to investigate the diagnostic potential of serum bile acid profiles as novel metabolic discriminators to differentiate among healthy individuals, CHB, LC, HCC, and liver failure, thereby addressing a key unmet need in clinical practice. **Methods:** A total of 625 participants were recruited and serum concentrations of 15 bile acids were determined by LC-MS/MS using targeted absolute quantification. Machine learning was employed to establish the diagnostic panels for classifying the distinct stages of HBV-related diseases. **Results:** The combinations of taurolithocholic acid (TLCA) and taurochenodeoxycholic acid (TDCA) effectively differentiated healthy individuals from the patients with liver diseases (AUCs = 0.880–1.000 across subgroups), and the specific panel of four bile acids achieved discriminative AUCs of 0.874 between CHB and LC, and 0.825 between CHB and HCC, which outperformed conventional biomarkers. Bile acid profiles also demonstrated significant responsiveness to antiviral therapy, some bile acid concentrations consistently decreasing during the post-treatment periods. **Conclusion:** Serum bile acid panels thus offer a sensitive and reliable diagnostic performance that could significantly enhance clinical decision-making and patient management.

## 1. Introduction

Liver disease is a serious public health concern with a high incidence and mortality. The primary etiological factors of liver disease include HBV, hepatitis C virus (HCV), alcohol abuse, obesity, and exposure to toxic substances [1,2,3], with HBV being the most critical contributor [4,5]. Chronic hepatitis due to HBV infection can progress to severe liver conditions, such as liver cirrhosis (LC), hepatocellular carcinoma (HCC), and liver failure (LF) [6,7]. The diagnosis of HBV-related disease subtypes is critically important for clinical practice. Organ image analysis is sometimes restricted by minor morphological changes in subtypes of liver disease, and biopsy is inherently invasive and relevant to secondary injuries [8]. Consequently, there is an urgent need for noninvasive blood biomarkers that provide comprehensive assessments of disease states related to HBV infection [9,10].

Serological tests for evaluating liver function enzymes are commonly employed in diagnosing liver disease. Although alanine aminotransferase (ALT) and aspartate aminotransferase (AST) serve as specific biomarkers reflecting liver injury severity [11,12,13], accumulating evidence indicates that patients with CHB, LC, and HCC may have normal ALT levels [14,15]. Therefore, these two biomarkers cannot reliably indicate the stages of disease progression. Lai et al. reported that the AST and ALT were prospective risk predictors for LC- in HBV-infected patients, with an area under the receiver operating characteristic curve (AUC) of 0.654 [16]. The advancement of metabolomics technology has significantly bolstered the study of liver disease biomarkers beyond conventional protein indicators. Cai et al. proposed that the combination of glycine and other metabolites distinguish liver disease patients, with an AUC of 0.753 for CHB versus healthy controls [17]. However, the challenge of accurately distinguishing HBV-related diseases through a single blood test remains unresolved in clinical practice.

Bile acids, synthesized, secreted, and metabolized in the liver, serve as important indicators of liver functions [18,19,20]. Total bile acid (TBA) concentration in blood has long been recognized as a significant indicator of liver function; however, TBA is ineffective for discriminating various states of liver diseases caused by HBV infection [21]. With the technological development of mass spectrometry, many bile acids have been identified in the serum [22]. Growing evidence suggests that specific bile acids in serum can act as diagnostic and predictive biomarkers for liver diseases [23]. Khalil et al. reported elevated levels of five conjugated bile acids in HCV-related HCC patients compared to noncirrhotic patients, with acceptable discrimination performance (AUC: 0.85–0.96) [24]. Despite the recognized importance of bile acids in liver diseases management, the specific bile acids suitable for diagnosis and their sensitivity to different liver diseases remain unclear. In our opinion, three aspects warrant priority in this field. First, when multiple etiologies coexist, the specific etiological background needs to be defined to clarify the correlation between liver disease symptoms and bile acid levels. Second, in clinical testing, determining bile acids with stable serum signals through mass spectrometry is crucial for elucidating their relationship with liver diseases. Third, while bile acid profiling employs a data-dependent acquisition mode in mass spectrometry, it may not suffice for distinguishing liver disease. Accurate quantification via mass spectrometry is crucial to clarify the roles of bile acids in liver disease diagnosis. To address these concerns, this investigation systematically examines liver diseases diagnosed as HBV infection, and targeted metabolomics via mass spectrometry was deployed to quantify bile acids in serum.

The study involves four key steps: (1) establishing a quantitative method using mass spectrometry to target bile acids in serum; (2) setting ALT and AST as references to compare the predictive performance of serum bile acid levels between healthy donors and HBV-related disease patients; (3) constructing machine learning models dependent on combinations of quantitative signals of bile acids aimed at discriminating different various HBV-related diseases; and (4) exploring the dynamic behavior of serum bile acids in patients with HBV-related liver disease following medical treatment. This investigation is expected to provide a set of convincing data that elucidate the roles of serum bile acids in recognizing the molecular differences among the subtypes of HBV-related diseases.

## 2. Materials and Methods

### 2.1. Cohort Design for HBV-Related Liver Diseases

As mentioned above, studying bile acids related to liver diseases is necessary to clearly define the specific etiological factors. In this study, HBV was regarded a unique etiological inducer for liver diseases. The patients suffering from liver diseases related to another hepatitis virus, alcoholic liver diseases, hereditary metabolic liver diseases, autoimmune liver diseases, pregnancy, lactation, and so on were excluded in this study. A total of 625 participants were enrolled from the Third People’s Hospital of Shenzhen in this study. The patient cohort consisted of 580 individuals with HBV-related liver diseases, comprising 4 groups, CHB, 175; LC, 174; HCC, 162; and LF, 69. Diagnoses were made based on established criteria: CHB was defined by hepatitis B surface antigen positivity for over six months; LC and HCC diagnoses adhered to the Chinese Guidelines for the Prevention and Treatment of Chronic Hepatitis B; and LF was assessed by the Chinese Group on the Study of Severe Hepatitis B (COSSH)-ACLF criteria. As a reference, 45 healthy donors were recruited, who matched the following criteria: (1) absence of hepatic pathology through clinical evaluation; (2) normal ranges for key biochemical markers (ALT, AST, TBA); and (3) exclusion if any clinical parameter which exceeded twice the upper limit of normal. For further assessments, if bile acid profiles worked as the prognosis indicators of medical treatments to HBV-related diseases, in total, 243 patients were collected, comprising 56 CHB, 56 LC, 78 HCC, and 53 LF. The longitudinal treatment analysis for those patients was based on the following criteria, in which (1) all patients were hospitalized at the Third People’s Hospital of Shenzhen; (2) none had received antiviral treatment prior to hospitalization, regardless of HBV-related liver disease subtype; (3) all patients received antiviral therapy, including entecavir, tenofovir disoproxil fumarate, or tenofovir alafenamide fumarate; and (4) the interval between the first and second serum collection were as close as possible, generally approximately 15 days.

All the participants provided informed consent, and the study protocol was approved by the Institutional Review Board of Ethics in BGI (Approval No. BGI-IRB-21020) and carried out according to the Declaration of Helsinki of the World Medical Association.

### 2.2. Preparation of Bile Acids from Serum

One volume of each serum sample was mixed with three volumes of cold precipitation reagent (methanol/acetonitrile (3/7, *v*/*v*), followed by strong vortexing and incubation at −20 °C for 20 min to precipitate proteins. The mixture was centrifuged at 10,000× *g* for 10 min at 4 °C, and the supernatant was utilized for bile acid analysis.

### 2.3. Bile Acid Measurement via LC–MS/MS

Potential bile acids in serum were selected based on published documents [22,25,26]. For quantification, 15 bile acid standards, including cholic acid (CA), chenodeoxycholic acid (CDCA), deoxycholic acid (DCA), lithocholic acid (LCA), ursodeoxycholic acid (UDCA), glycocholic acid (GCA), glycochenodeoxycholic acid (GCDCA), glycodeoxycholic acid (GDCA), glycolithocholic acid (GLCA), glycoursodeoxycholic acid (GUDCA), taurocholic acid (TCA), taurochenodeoxycholic acid (TCDCA), TDCA, TLCA, and tauroursodeoxycholic acid (TUDCA), were purchased from Steraloids Inc. (Newport, RI, USA). Deuterium-labeled bile acids, such as d4-DCA, d4-LCA, d4-GDCA, and d4-GCA, were also obtained from the same supplier. Standards and stable isotope-labeled standards were prepared in methanol as individual stock solutions at a 1 mM concentration and stored at −80 °C. The internal standard concentrations were held constant across calibration points.

Bile acids were separated by Waters ACQUITY UPLC BEHC18 column (2.1 mm× 5 cm, 1.7 µm, 130 Å) installed in Waters ACQUITY UPLC I-Class System (Waters, Milford, MA, USA) at a flow rate of 0.5 mL/min with a gradient (Table S1). The BA profile was analyzed by the Quad 6500 mass spectrometer (SCIEX, Framingham, CA, USA) operated under the MRM mode.

Stable isotope-labeled internal standards of bile acids were purchased from BePure (Beijing, China) and were spiked into all samples and QC samples that were made by pooled serum. A QC sample was run every 10 samples for bile acids analysis by LC-MS/MS followed by correction of retention time and MS/MS intensity between the sample and QC. After several running batches, the bile acids in the QC samples were quantified by the individual bile acid calibration curves. The concentration median of individual bile acids in the QC samples were further used for global normalization and the estimation of bile acids in all samples. A batch was flagged for reanalysis if a QC sample deviated beyond the $\bar{X}$ ± 3SD range or if two QCs exceeded the $\bar{X}$ ± 2SD.

### 2.4. Data Processing of the Quantitative Bile Acid Contents in the Samples

The acquired raw mass spectrometry data were processed via Multiquant™ software 3.0.3 (SCIEX, Framingham, CA, USA), including the construction of calibration curves, determination of retention time windows, and extraction of target chromatographic peaks.

The quantitative bile acid data were processed in two steps: (1) exclusion of samples with >20% missing values and (2) imputation of the remaining missing values (attributable to a low signal response) at half the minimum concentration detected per analyte in all the samples.

### 2.5. Statistical Analysis

Quantitative bile acid data were evaluated for normality using the Shapiro–Wilk test in the R package (version 4.2.1, Lucent Technologies, Murray Hill, NJ, USA) “stats”. If normally distributed, Student’s *t* test was used; otherwise, the Wilcoxon test was used. Comparison between healthy controls and patients with different types of HBV-related liver diseases or among various liver diseases were performed in an unpaired mode, while treatment efficacy assessments employed a paired mode. All statistical significance assessments were adjusted for multiple comparisons via the Bonferroni method, with corrected *p*-values reported. For introducing an integrated function that combines the bile acids sensitively responding to therapeutic treatments, a predefined rule-based approach was used, in which a bile acid with the largest concentration response after treatment in all possible bile acids sensitive to drugs was selected to a fixed weighting scheme, and a new function, termed ${BA}_{i}$, was achieved by the aggregation of weighted bile acids from pre/post-treatment [27] (the estimation formulas in detail are in Supplementary Methods).

### 2.6. Model Construction and Assessment

The main program and models were implemented in Python (version 3.11, Python Software Foundation) using open-source packages, including package Scikit-Learn (version 1.4.2), NumPy (version 2.2.6), Pandas (version 2.3.0), Matplotlib (version 3.8.4), imblearn-learn (version 0.11.0). To identify robust bile acid profiles for paired comparisons discrimination, we developed a systematic machine learning pipeline encompassing data balancing, feature selection, and rigorous independent validation. The dataset was randomly partitioned into a 70% training set and a 30% independent testing set using stratified sampling. To address imbalanced sample sizes, the synthetic minority oversampling technique was applied exclusively to the training set. The independent test set remained untouched, preserving its original biological distribution to ensure an unbiased evaluation of model generalizability. Five machine learning algorithms that are commonly taken in biology study, like random forest, support vector machine (SVM), k-nearest neighbors (KNN), extreme gradient boosting (XGB), and gradient boosting decision tree (GBDT), were first selected to evaluate the best performance on a representative dataset from this study. The performance method, such as discriminative power and running stability, was carefully assessed in the benchmark in parallel, and the method with the best performance was selected as the core algorithm for all subsequent modeling tasks. The selection of bile acid features using GBDT was divided to two phases. (1) All the individual bile acids were statistically assessed for their concentration differences among the groups, healthy versus patients or patients versus patients. The bile acids with significant concentration changes were selected as the feature candidates. (2) All possible feature combinations were put into an exhaustive modeling in GBDT to seek the best discrimination with a minimum set of bile acids among the groups interested. To prevent information leak during discriminator construction, hyperparameter space exploration was taken in a 10-fold stratified cross-validation scheme within the training, in which Bayesian optimization was utilized to prevent over-fitting. After model construction, a discriminator was further validated with permutation in the independent test set.

## 3. Results

### 3.1. Clinical Characteristics of the Participants

This study enrolled a cohort of 625 donors, whose demographic characteristics are detailed in Table 1. Male predominance was observed in both the healthy control group and HBV-related disease subgroups (60%, 70%, 82%, 88%, and 83%, respectively). Biochemical analyses revealed significant intergroup differences in ALT, AST, and TBA levels (*p* < 0.0001), indicating molecular heterogeneity in hepatic injury across distinct disease states. Notably, the pronounced disparity in TBA among groups underscores the necessity for further investigation into disease-specific alterations in bile acid profiles.

### 3.2. Method for Bile Acid Quantification in Serum

**Figure 1:**
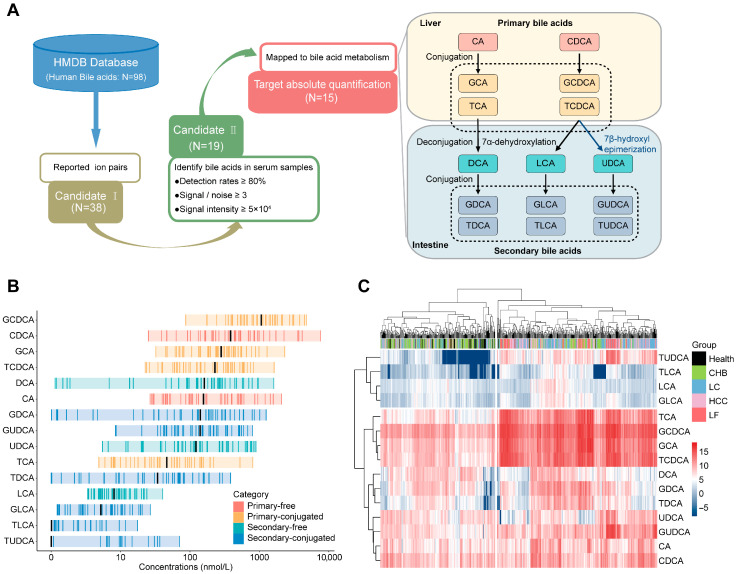
Establishment of bile acid candidates in serum identified by LC–MS/MS and preliminary profiles of serum bile acids in healthy individuals and patients with HBV-related diseases. (**A**) Selection of the serum bile acids detectable by LC–MS/MS. (1) Based on database of HMDB, 98 bile acids have been collected, while only 38 bile acids have been documented by their identification with mass spectrometry with clear information of ion pairs. (2) The ion pairs of 38 bile acids were employed as parameters for MRM analysis of serum samples from a healthy cohort to access identification rates and MRM signal intensities. A total of 19 bile acids were detected, achieving over 80% detection rates with intensities ≥ 50,000 and signal-to-noise ratios > 3. (3) Upon well recognized bile acid metabolic pathways, four bile acids were excluded due to insufficient biochemical significance. (**B**) Concentration ranges of the 15 bile acids in healthy serum; black bars represent the median concentration of each bile acid. (**C**) Hierarchical clustering of the concentrations of 15 bile acids against HBV-related liver diseases and health.

Through the filtration process targeting stable serum bile acids, 15 compounds were ultimately selected as analytical targets (Figure 1A). Optimization of the LC–MS/MS system in the MRM mode was performed to enhance the quantification of selected bile acids in the serum, with the optimized ion pair parameters and elution gradient of liquid chromatography summarized in Tables S1 and S2. The optimized ion chromatogram for the bile acid standards is shown in Figure S1A; these bile acids are well separated and identified under such conditions. Calibration curves for bile acid standards were generated in bile acid-free serum, yielding linear equations, as shown in Table S3, in which all the correlation coefficients exceeded 0.99, with large dynamic concentration ranges within 10^3^. The accuracies of all bile acids were between 85% and 115%, and the precisions were less than 10% for all levels tested (Table S4). Through method optimization, quantitative calibration and recovery examination, the bile acids in the serum were quantitatively measured with good quality.

Hierarchical clustering of healthy cohort data (*n* = 45) segregated bile acids into lower variation (TDCA, LCA, TLCA, GLCA, and TUDCA) and the higher variation (Figure S1B). Moreover, the bile acid concentrations in healthy serum are presented in Figure 1B, revealing that primary bile acid generally exceeded secondary counterparts in median concentration (exceptions: CA and TCA) and primary and 56% of secondary bile acids displayed over 100-fold concentration dynamics, contrasting with narrow ranges (<100-fold) in others. The distribution of the biochemical and abundant properties of bile acids in the serum provides valuable information for the classification of liver diseases.

An analysis of 625 serum samples of healthy donors and HBV-related liver diseases via hierarchical clustering (Figure 1C) showed the following: (1) The samples are divided into two clusters: the left cluster (93% healthy donors, 62% CHB) and the right cluster (97% LF, 67% HCC, 68% LC), and this distribution suggests that serum bile acid profiles in healthy donors differ substantially from those in LF, HCC, and LC patients, but are similar to those in CHB patients; (2) Near-complete segregation of healthy donors and LF patients, supporting bile acid as indicators of normal liver and liver failure; and (3) Inability to distinguish disease subtypes by global concentrations, necessitating subtype-specific biomarker selection. Therefore, overall concentration analysis of serum bile acids is potentially valuable in the molecular diagnosis of various HBV-related liver diseases.

In addition, the potential impact of gender and age on serum bile acid profiles was evaluated in healthy donors. Correlation analysis revealed no significant association of gender or age with the concentrations of serum bile acids (Figure S1C,D).

### 3.3. Serum Bile Acids as Candidates for Identifying Differences Between Healthy Individuals and Patients with HBV-Related Diseases

To construct discriminators for health and HBV-related diseases based on serum bile acids, the discriminative efficiency of individual bile acids was first evaluated, then several candidates were selected based on their ability to discriminate between health and HBV-related diseases, and combinations of minimized bile acid candidates were assessed to construct discriminators with better performance.

**Figure 2:**
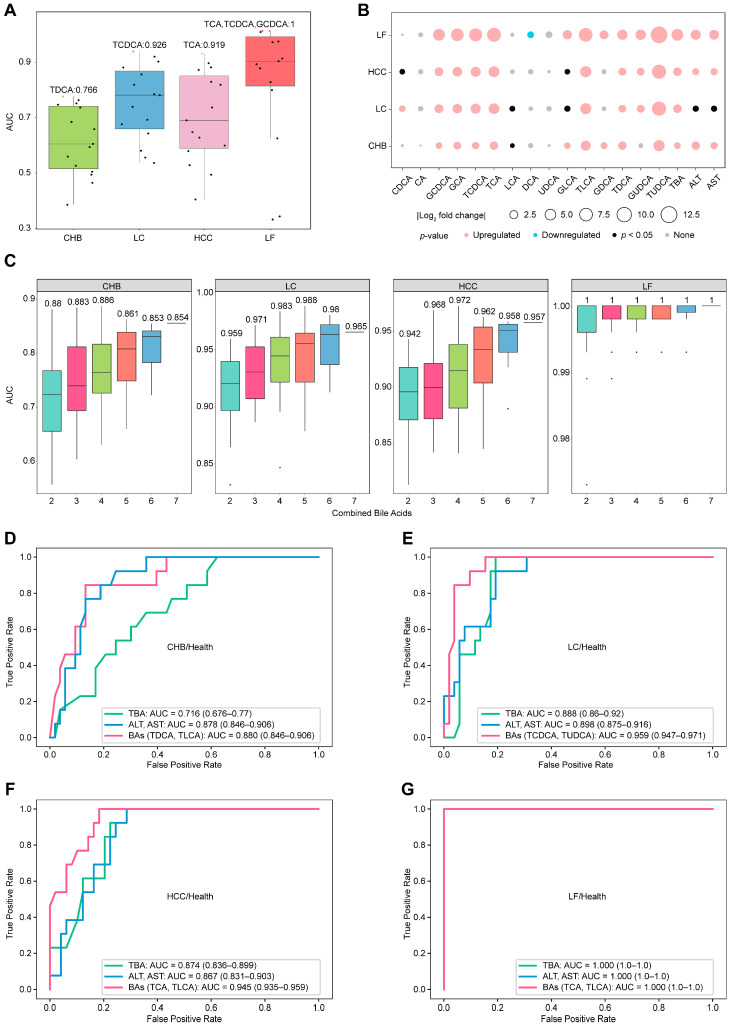
Differential concentration analysis and discrimination of serum bile acids between healthy individuals and patients with HBV-related diseases. (**A**) Average AUC values of 15 individual bile acids for distinguishing health status and disease status via GBDT. Individual AUC values are shown as points, with the maximum value highlighted in color. (**B**) Analysis of the differences in the concentrations of individual bile acids with respect to health and disease. Upregulated: *p* < 0.05, fold change (FC) > 2; downregulated: *p* < 0.05, FC < 0.5; none: *p* > 0.05. (**C**) AUC distributions for the cumulative combinations of bile acids used to distinguish health and disease status via GBDT, with the numbers indicating the maximum average AUC values for each combination. (**D**–**G**) Discriminator ROCs of the combined bile acids to distinguish health and diseases with ALT, AST, and TBA as the references.

The average AUC for each single bile acid demonstrated basic discrimination capability between healthy donors and four different subtypes of HBV-related liver diseases in Figure 2A, although the separation from CHB patients was limited. These observations basically agree with the results presented in Figure 1C, in which the serum concentrations clustered healthy individuals and CHB patients together, while effectively distinguishing other liver disease subtypes. The statistical analysis in Figure 2B showed that, while not all free bile acids exhibited significant differences, most primary-conjugated bile acids and conventional serum markers (ALT, AST, TBA) did differ significantly between healthy controls and liver disease patients. Among these bile acids, seven, such as GCDCA, GCA, TCDCA, TCA, TLCA, TDCA, and TUDCA, were selected (*p*-value < 0.05 and |log_2_-fold change| > 1). Exhaustively enumerating all combinations of the seven bile acids yielded 120 combinations. The average AUC values for different combinations of bile acids are presented in Figure 2C, implying that increasing the number of bile acids in a combination enhances discriminative efficiency for health versus liver diseases, whereas combinations of two bile acids demonstrated significantly improved efficacy compared to single bile acids. The combinations yielding the highest AUCs were selected as the biomarkers for distinguishing health from liver diseases (Figure 2D–G): TDCA and TLCA for health/CHB (AUC = 0.880, (95% confidence interval (CI): 0.864–0.906)), TCDCA and TUDCA for health/LC (AUC = 0.959 (CI: 0.947–0.971)), TCA and TLCA for health/HCC (AUC = 0.945 (CI: 0.935–0.959)), and 61% of the combinations with two bile acids for health/LF (AUC = 1 (CI: 1–1)).

Furthermore, we evaluated the ability of bile acid combinations to outperform traditional biomarkers such as ALT, AST, or TBA in recognizing health versus liver diseases. All biological measurements were obtained from the same cohort samples, and the same machine learning model was employed. Figure 2D–G summarize all the comparison results with combinations of bile acids versus traditional biomarkers to discriminate health from liver diseases. The combination of AST and ALT or TBA achieved acceptable AUC values for distinguishing health from disease, with AUC values correlating positively with the severity of liver disease from hepatitis to cirrhosis, carcinoma, and failure. Selected two-bile acid combinations consistently improved recognition efficiency, yielding increased AUC values across individual comparisons. Among the comparisons, the combination of TDCA and TLCA or the combination of AST and ALT is good enough to differ in terms of health status from CHB, with an AUC of 0.880 or 0.878, whereas TBA is relatively weak in terms of discrimination, with an AUC of 0.716. Moreover, the AUC values of TDCA and TLCA for the other three recognition methods (health/HCC, health/LC and health/LF) were obviously higher than those for health/CHB, with AUC values of 0.913, 0.926, and 0.989, respectively. In conclusion, TDCA and TLCA levels in serum constitute a set of biomarkers that can be used to distinguish health status from HBV-related liver disease globally.

### 3.4. Serum Bile Acids as Candidates for Identifying Differences Among HBV-Related Diseases

**Figure 3:**
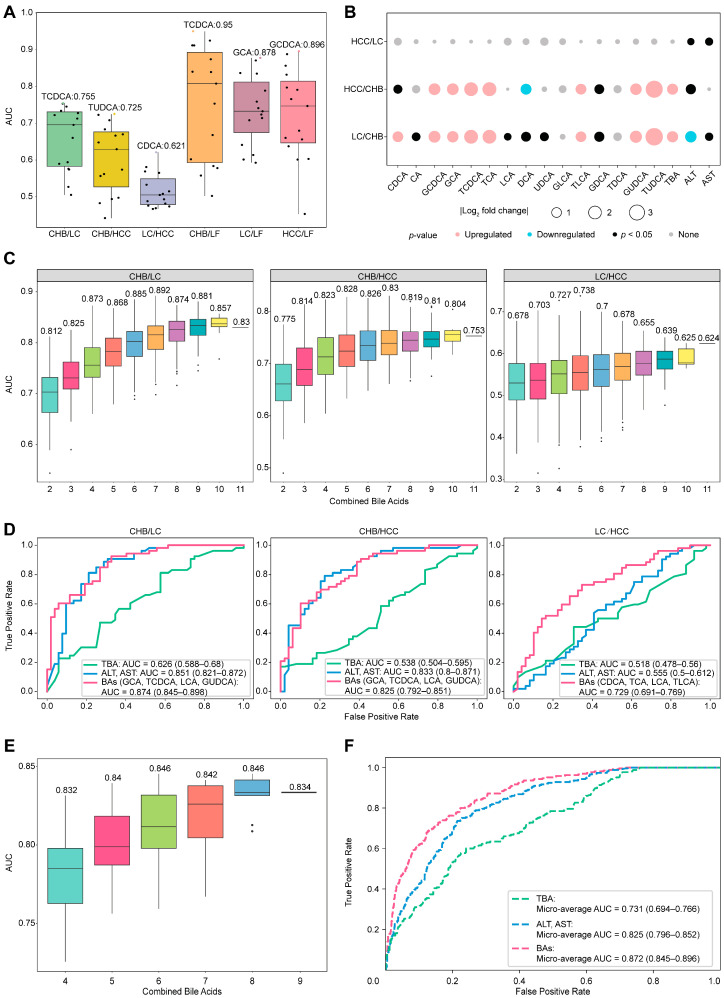
Discriminator construction for any 2 HBV-related liver diseases based on individual and combined bile acids. (**A**) Average AUC values of 15 individual bile acids for distinguishing any two diseases via GBDT. Individual AUC values are shown as points, with the maximum value highlighted in color. (**B**) Analysis of the differences in the concentrations of individual bile acids between any two diseases: upregulated: *p* < 0.05, fold change (FC) > 2; downregulated: *p* < 0.05, FC < 0.5; and none: *p* > 0.05. (**C**) AUC distributions for the cumulative combinations of bile acids used to distinguish between any two diseases via GBDT, with the numbers indicating the maximum AUC values for each combination. (**D**) Discriminator ROCs of the combined bile acids to distinguish any two diseases with ALT, AST, and TBA as the references. (**E**) The AUC distribution for bile acid combinations to create a general panel that is capable of distinguishing between any two groups in healthy individuals and patients with HBV-related diseases. (**F**) Micro-average ROCs generated with a multiclassification model with a selected panel with six bile acids, in which ALT, AST, and TBA serve as references in the same cohort.

Serum bile acids were further evaluated as indicators to distinguish HBV-related liver diseases subtypes. Employing a strategy consistent with prior analyses, mean AUC values of individual bile acids were stratified into three tiers (Figure 3A): (1) good discriminators (AUC ≥ 0.8) in CHB/LF, LC/LF, and HCC/LF; (2) moderate discriminators (AUC 0.7–0.8) in CHB/LC and CHB/HCC; and (3) poor discriminators (AUC ≤ 0.7) in LC/HCC. Single bile acids effectively distinguished LF from others, but showed limited sensitivity in differentiating CHB, LC, and HCC, necessitating the investigation of bile acid combinations for enhanced discrimination.

Significant differences in the serum concentrations of single bile acid between the two subtypes of HBV-related liver diseases were estimated and are listed in Figure 3B. A total of ten bile acids, CDCA, GCDCA, GCA, TCDCA, TCA, DCA, TLCA, GDCA, GUDCA, and TUDCA, exhibited significantly different serum concentrations (*p* < 0.05) between CHB/LC and CHB/HCC, except for LC/HCC. AST and ALT are not acceptable indicators of differences for these comparisons, whereas TBA may differ for CHB/LC or CHB/HCC but still fails in LC/HCC. To select bile acid candidates for combination analysis via GBDT, these ten bile acids were initially selected to discriminate any comparisons. Considering the difficulty in distinguishing LC from HCC, LCA was included in the discriminator construction with AUC over 0.55. Following this, all combinations of the selected bile acids were exhaustively enumerated for the discrimination of these subtypes (Figure 3C), in which a combination with two more bile acids always achieves better differentiation performance, and those with combinations of four bile acids reach stable efficiencies in differentiating all pairwise disease comparisons. Compared to single bile acids, combinations of four bile acids enhanced the discrimination: from moderate levels to good levels for CHB/LC and CHB/HCC, and from poor to moderate for LC/HCC. Based on Figure 3D, the optimal panels identified were as follows: GCA, TCDCA, LCA, and GUDCA for CHB/LC (AUC = 0.874 (CI: 0.845–0.898)) and CHB/HCC (AUC = 0.825 (CI: 0.792–0.851)); and CDCA, TCA, TLCA, and LCA for LC/HCC (AUC = 0.729 (CI: 0.691–0.769)). Intriguingly, two bile acids (TDCA-TLCA) can globally distinguish health from HBV-related liver disease. The composition of each panel with four bile acids for differentiating any two disease states varies considerably, suggesting that bile acid alteration is sensitive to liver disease status.

Compared to traditional biomarkers (Figure 3D), ALT and AST exhibit comparable discriminations to CHB/LC (AUC = 0.851 (CI: 0.821–0.872)) and CHB/HCC (AUC = 0.833 (CI: 0.8–0.871)) but fail to distinguish LC/HCC (AUC = 0.555 (CI: 0.5–0.612)); moreover, TBA globally displays poor discrimination for all comparisons. Importantly, the discrimination efficiencies of the combined bile acids in these comparisons are globally better than those judged by ALT-AST or TBA, implying that bile acid profiles in serum possess obvious advantages in providing molecular evidence differing between any two subtypes of HBV-related liver diseases. Thus, the question remains as to whether a general panel of combined bile acids could be used for discrimination for this purpose.

The GBDT one-vs-one multi-classification algorithm was used to identify the optimal combinations of nine bile acids (TUDCA, TDCA, CDCA, TCDCA, GCA, TCA, GUDCA, TLCA, and LCA) derived from the union of all discriminators, enabling the differentiation of all physiological statuses. As indicated in Figure 3E, discrimination performances for these groups based on various bile acid combinations were comparably and consistently high, whereas the combination of six bile acids appeared to reach a saturation point. When compared to traditional biomarkers, the final bile acid panel (GCA, CDCA, TCDCA, TDCA, LCA, and TLCA) demonstrated superior classification performance (micro-average AUC = 0.872 (CI: 0.845–0.896)) across all physiological statuses, as presented in Figure 3F. However, the accuracy was not satisfactory when the confusion matrix was checked (Figure S2). Although some combinations of bile acids work well for distinguishing between any two types of HBV-related liver disease, the development of a universally applicable bile acid panel remains a challenge.

### 3.5. Evaluation of the Changes in Bile Acid Levels in the Serum of HBV-Related Patients in Response to Chemical Therapy

**Figure 4:**
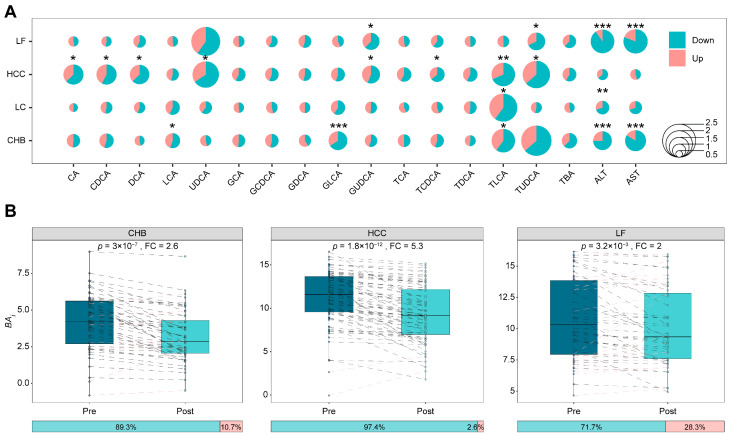
The responses of bile acid concentrations in different HBV-related liver diseases to pre- and post-TDMI treatment. (**A**) Bubble plot reflecting the changes in the concentrations of bile acids before and after antiviral treatment in patients with different diseases that were statistically significant. * *p* < 0.05, ** *p* < 0.01, *** *p* < 0.001. (**B**) Boxplots show pre- and post-treatment distributions of patient-level integrated bile acids index, with paired lines indicating index response in each patient. In these paired lines, black represents a decrease post-treatment, while red represents an increase. The horizontal stacked bar below each boxplot shows the proportion of patients exhibiting a decrease (left) or increase (right) in the representative bile acid after treatment.

To assess whether bile acid concentration in HBV-related patients responds to antiviral treatment, a total of 243 paired serum samples were collected from patients after 1–2 weeks of antiviral treatment, comprising 56 CHB, 56 LC, 78 HCC, and 53 LF patients. Changes in individual bile acid concentrations from pre- to post-treatment were assessed for significance using paired tests (*p* < 0.05) across the four HBV-related diseases; the analysis assessed the magnitude of change (fold change ≥ 1.2) and the direction of concentration changes (increase or decrease), requiring over 55% of paired samples to reflect the main trend. The overall response of the 15 bile acids to antiviral treatment in the four HBV-related diseases is elucidated in Figure 4A, revealing that the concentration changes and direction changes seem typically disease-dependent. Bile acids whose concentration responded to antiviral treatment included LCA, GLCA, and TLCA in CHB; TLCA in LC; CA, CDCA, DCA, UDCA, GUDCA, TCDCA TLCA, and TUDCA in HCC; and GUDCA and TUDCA in LF. The concentrations of ten bile acids in patient serum are highly sensitive to antiviral therapy; notably, they were previously identified as biomarkers to discriminate healthy liver from liver disease or between liver diseases (Figure 2 and Figure 3). Moreover, concentrations of all bile acids sensitive to antiviral treatment decreased, implying that antiviral therapy partially improve bile acid metabolism. Serum ALT and AST levels were measured as references, with data in Figure 4A showing a significant decrease in their levels following antiviral treatment, except in HCC, which supports the conclusions drawn from bile acid concentration changes. Therefore, bile acids not only serve as biomarkers for HBV-related liver diseases but also indicate therapeutic responses in patients.

A single bile acid concentration is susceptible to the individual metabolism status, thereby affecting the judgment of the therapeutic effect; however, combination information from the concentrations of multiple bile acids may address this issue. As noted, ten bile acids were responsive to antiviral treatment across different HBV-related diseases. For overall estimation of serum bile acid in response to the therapy efficiency in these patients with liver diseases, the function termed as ${BA}_{i}$ onto each bile acid was introduced. As mentioned in “Materials and Methods”, ${BA}_{i}$ integrates the bile acids whose serum concentrations are deceased in maximum in responding to the therapy and represents the weighted signals of combined bile acids. Figure 4B exhibits the comparison of ${BA}_{i}$ values between pre- and post-antiviral therapy in three HBV-related diseases, CHB, HCC, and LF, revealing that the patient coverage due to such therapy is improved from 66.1%, 66.7%, and 67.9% to 89.3%, 97.4%, and 71.7% with fold changes of ${BA}_{i}$ at least 2, respectively. The integration of multiple bile acids with weighted concentrations, therefore, is helpful for a better evaluation of antiviral efficacy.

## 4. Discussion

We systematically analyzed the characteristic changes in the serum bile acid profiles of patients with HBV-related liver diseases in this study, revealing that combinations of serum bile acids can serve as sensitive panels of biomarkers for the discrimination of these diseases. On the basis of the accurate quantification of serum bile acids and the evaluation of discrimination models, the conclusions of this study are as follows: (1) Individual serum bile acids could not differ across HBV-related liver diseases globally, whereas the combination of TDCA and TLCA could distinguish healthy donors well from all diseases groups; (2) among seven bile acids, CDCA, TCDCA, GCA, TCA, GUDCA, TLCA, and LCA, combinations of four could reliably differentiate any two subtypes; and (3) these bile acids not only work as biomarkers of health from liver disease or among subtypes but also function as therapeutic indicators of antiviral drugs. These conclusions benefit from the systematic quantitative assessment of bile acid profiles and the strict selection of liver diseases caused by a single etiological factor, HBV. Moreover, many observations of this study are not unequal findings but are supported by other previous reports, even though those studies were not specifically focused on HBV-related conditions. For example, Sun et al. reported increased conjugated bile acids in CHB patients with an AUC below 0.8 for single bile acid discrimination models [28]. In a profiling study of serum bile acids, Wang et al. reported that a single bile acid appeared relatively poor at discriminating between HBV-related LC and CHB; however, a panel consisting of platelet, TUDCA, UDCA, TLCA, LCA, and CA showed a strong discrimination ability for these two diseases, with an AUC of 0.89 [29]. Moreover, Wu et al. perceived that the use of ALT and AST as biomarkers was not sensitive enough to distinguish LC from HCC, suggesting bile acid alterations in these two liver diseases [30]. In contrast to previous investigations on bile acids in liver diseases, as mentioned in the “Introduction”, we adopted a novel strategy that specifically focused on a single etiological cause, globally profiled the stable bile acids in serum, accurately quantified bile acids, and achieved new findings regarding the use of serum bile acids as good biomarkers of liver diseases.

Several investigators have examined the relationship between bile acid forms and liver diseases. For instance, Xun et al. reported a significant increase in conjugated bile acids in CHB patients compared with healthy individuals but no difference in free bile acids between the groups [31]. Similarly, Luo et al. observed elevated concentrations of conjugated bile acids in patients with HCV-related liver disease but not in healthy individuals [32]. With respect to this issue, our study revealed three notable findings: (1) the serum concentrations of conjugated bile acids were more sensitive than free bile acids when comparing healthy individuals with those suffering from HBV-related liver diseases; (2) alterations in conjugated bile acid levels correlated with disease severity, with the most pronounced changes observed in LF; and (3) some conjugated bile acids, such as TLCA, serve as biomarkers for therapeutic response, displaying significantly reduced serum levels following antiviral treatment. It is well known that the sodium taurocholate cotransporter peptide (NTCP) on the basement membrane of liver cells mediates the uptake of bound bile acids and functions as a receptor for HBV invasion [33]. HBV and bile acids, therefore, can competitively bind to NTCP. After HBV binds to NTCP, the uptake of bile acids by liver cells is restricted, thereby promoting the compensatory synthesis of bile acids and their conjugates in the liver [34,35]. In addition, the immune response caused by HBV infection damages liver cells, resulting in a decrease in the number of NTCP and a decrease in the uptake and transformation ability of bile acids, leading to the increased synthesis of bile acids and related conjugates in the liver [36]. Our data provide a plausible mechanism that responds to HBV infection, and the conversion of conjugated bile acids is strengthened in all liver disease statuses. With respect to taurine conjugates, Thomas et al. proposed another mechanism in which the upregulation of Toll-like receptor 4 expression could change intestinal permeability and correspondingly enhance taurine conjugate uptake [37]. In short, the large disturbance in the concentrations of conjugated bile acids in different HBV-related liver diseases prompts a research direction in both diagnosis and therapeutics.

Despite the high performance of our diagnostic models, several limitations warrant consideration. First, comorbidities that may have an influence on discriminating with metabolic overlap, such as advanced fibrosis, can induce a dominant metabolic disturbance that eclipses malignancy-specific signals [24,38,39]. Second, while the gut-liver axis is known to profoundly shape bile acid profiles through microbial transformation [40,41,42], a systematic investigation into the longitudinal, microbiota-mediated dynamic changes in bile acids during HBV progression was not within the scope of this study. Finally, because there exist many subtypes of the HBV genome that are partially race- or geography-dependent [43,44,45], the generalizability of our findings across diverse populations remains to be fully established. To refine diagnostic resolution, future frameworks must integrate specific pathological stages into the modeling process to account for microenvironmental confounding. Furthermore, incorporating longitudinal microbiome analysis alongside bile acid profiling holds promise for revealing the mechanistic drivers of hepatic injury and identifying novel avenues for integrated diagnosis and therapeutic monitoring. Future efforts will prioritize large-scale, multicenter, blinded validation across diverse ethnic cohorts, to ensure the global generalizability and clinical utility of these bile acid signatures.

## 5. Conclusions

In conclusion, serum bile acid profiles serve as sensitive biomarkers for HBV-related liver diseases. The TDCA-TLCA panel robustly distinguishes healthy individuals from those with all HBV-related hepatopathies, whereas quadruple-bile acid combinations accurately differentiate disease subtypes. These profiles further predict antiviral therapy response, outperforming traditional biomarkers in diagnosis and therapeutic monitoring. By establishing a standardized LC–MS/MS quantification framework validated in 625 clinical samples, this study provides clinicians with a minimally invasive tool for the precise diagnosis of HBV-driven liver disease subtypes and real-time efficacy evaluation.

## Figures and Tables

**Table 1:** The clinical characteristic features of participants.

Variable	Health	CHB	LC	HCC	LF	*p*-Value
Donors	45	175	174	162	69	-
Female/Male	18/27	53/122	31/143	19/143	12/57	<0.0001 ^a^
Age (years)	48.44 ± 10.54	38.14 ± 9.68	48.97 ± 12.09	53.81 ± 11.74	48.91 ± 15.49	<0.0001 ^b^
AST (U/L)	18.22 ± 4.51	110.45 ± 216.04	44.19 ± 49.50	80.79 ± 140.99	410.24 ± 572.09	<0.0001 ^b^
ALT (U/L)	15.86 ± 7.55	198.33 ± 379.87	39.08 ± 61.57	56.47 ± 127.79	561.83 ± 716.99	<0.0001 ^b^
TBA (μmol/L)	3.99 ± 2.63	24.94 ± 56.04	39.94 ± 53.31	45.79 ± 80.58	203.51 ± 124.24	<0.0001 ^b^

^a^ χ2 test, ^b^ Kruskal–Wallis test.

## Data Availability

The authors confirm that the data supporting the findings of this study are available. Raw data that support the findings of this study are openly available in CNGBdb at https://db.cngb.org/ (URL accessed on 17 July 2025), reference number CNP0007708.

## Supplementary Materials

The following supporting information can be downloaded at https://www.mdpi.com/article/10.3390/biomedicines14010084/s1, Figure S1: Bile acids profiles in health samples; Figure S2: Multi-classification model evaluation; Table S1: Multiple reaction monitoring parameters for bile acids, the asterisk indicated the quantifier transition for each bile acid; Table S2: Elution gradient for bile acids separation; Table S3: Quantitative range and linearity for 15 bile acids; Table S4: The precision and average accuracy of 15 bile acids in LQC, MQC and HQC samples. Supplementary Methods: Definition of an integrated function that combines the bile acids responding to therapeutic treatments.
