# Supplementary material for: Serum Bile Acid Profiling Across the Full Spectrum of HBV-Related Liver Diseases in Chinese Population: Implications for Diagnosis and Treatment Assessment
Source: Biomedicines. 2025 Dec 31;14(1):84. doi: 10.3390/biomedicines14010084 (PMC12838154; doi:10.3390/biomedicines14010084)
Supplement: Supplementary file 1 [file biomedicines-14-00084-s001.zip › biomedicines-4034008-supplementary.pdf]

Supplementary

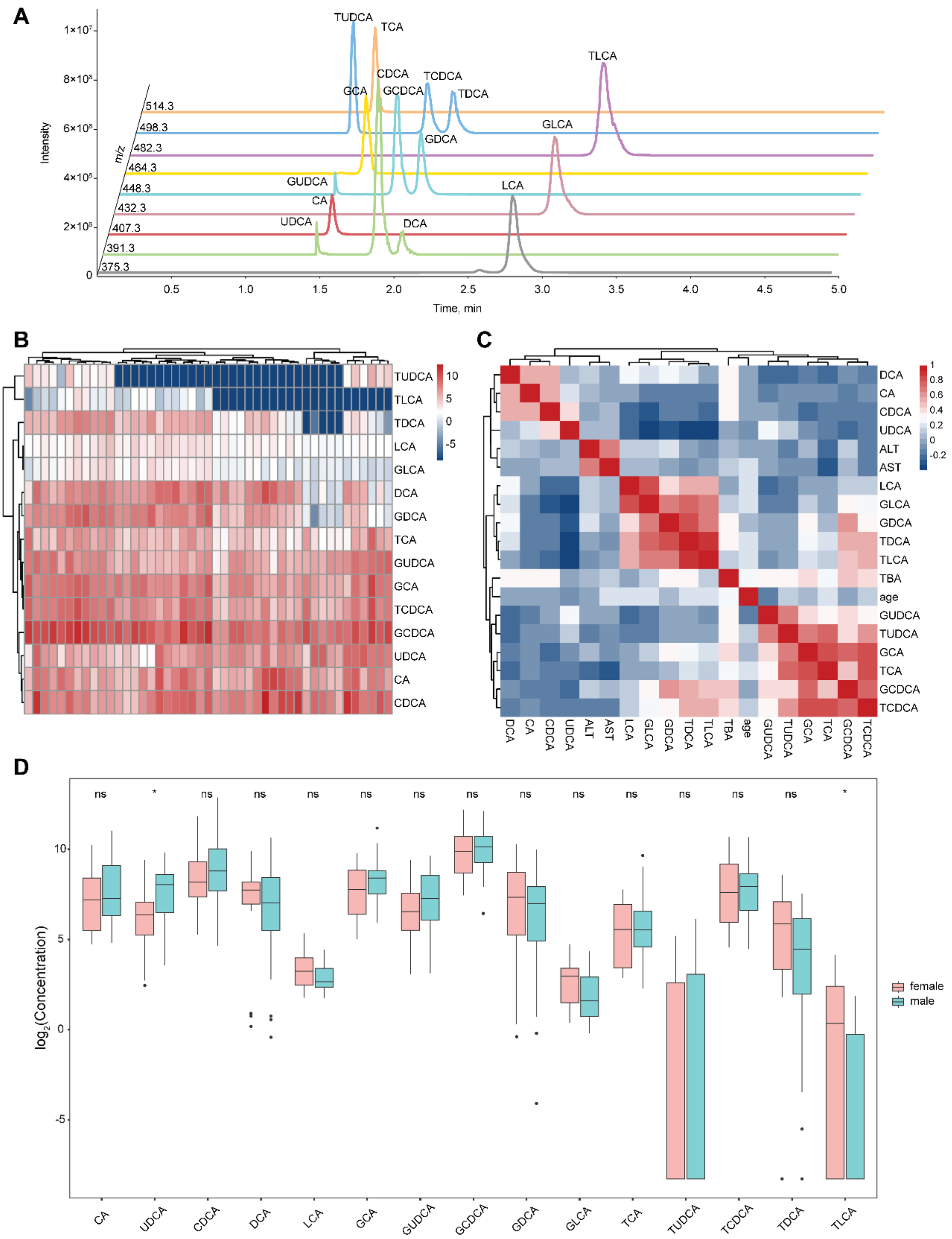

Figure S1. Bile acids in health samples. (**A**) The MRM chromatograms of 15 standard bile acids. (**B**) Hierarchical clustering heatmap of all health samples. (**C**) Correlation between age and bile acids. (**D**) Comparative analysis of serum bile acid profiles between male and female groups. ns, no significant, * $p < 0.05$.

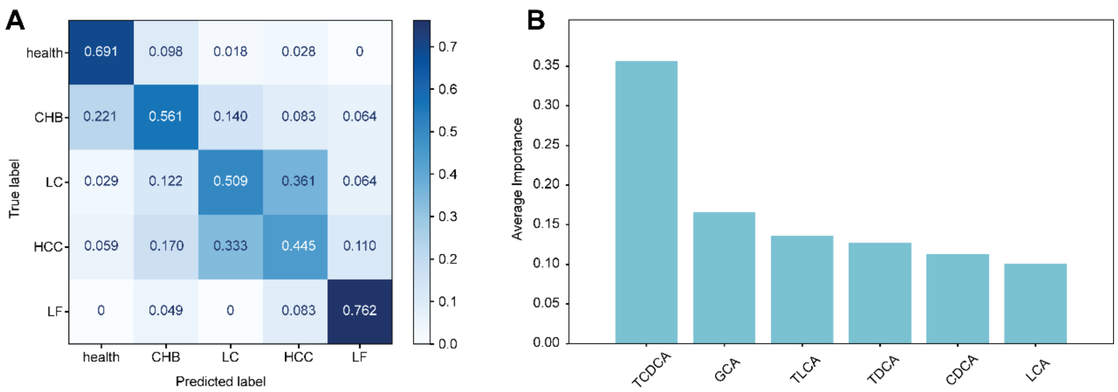

Figure S2. Multi-classification model evaluation. (**A**) Confusion matrix of classification. (**B**) Feature importance ranking of bile acids.

Table S1 Multiple reaction monitoring parameters for bile acids, the asterisk indicated the quantifier transition for each bile acid.

Compound name	Precursor ion (Da)	Product ion (Da))	Retention time (min))	Declustering Potential (volts)	Collision Energy (volts)	Collision Cell Exit Potential (volts)
CA	407.3	407.3	1.51	-130	-12	-13
CA*	407.3	343.3	1.51	-130	-46	-11
UDCA*	391.3	391.3	1.42	-125	-14	-3
CDCA*	391.3	391.3	1.84	-125	-14	-3
DCA*	391.3	391.3	2.00	-125	-14	-3
LCA*	375.3	375.3	2.78	-135	-15	-13
GCA	464.3	464.3	1.56	-120	-16	-29
GCA*	464.3	74.0	1.56	-120	-70	-3
GUDCA	448.3	448.3	1.46	-120	-14	-13
GUDCA*	448.3	74.0	1.46	-120	-64	-13
GCDCA	448.3	448.3	1.94	-120	-14	-13
GCDCA*	448.3	74.0	1.94	-120	-64	-13
GDCA	448.3	448.3	2.10	-120	-14	-13
GDCA*	448.3	74.0	2.10	-120	-64	-13
GLCA	432.3	432.3	2.94	-120	-14	-13
GLCA*	432.3	74.0	2.94	-120	-64	-13
TCA	514.3	514.3	1.64	-180	-18	-19
TCA*	514.3	80.0	1.64	-180	-105	-10
TUDCA	498.3	498.3	1.50	-100	-14	-10
TUDCA*	498.3	80.0	1.50	-180	-105	-10
TCDCA	498.3	498.3	2.05	-180	-14	-17

TCDCA*	498.3	80.0	2.05	-180	-105	-10
TDCA	498.3	498.3	2.23	-180	-14	-17
TDCA*	498.3	80.0	2.23	-180	-105	-10
TLCA	482.3	482.3	3.15	-175	-16	-17
TLCA*	482.3	80.0	3.15	-175	-104	-7
d4-DCA	455.3	395.3	2.00	-35	-14	-3
d4-DCA*	395.3	395.3	2.00	-125	-14	-3
d4-LCA	439.3	379.3	2.78	-40	-32	-11
d4-LCA*	379.3	379.3	2.78	-135	-12	-13
d4-GDCA	452.3	452.3	2.10	-120	-12	-15
d4-GDCA*	452.3	74.0	2.10	-120	-64	-5
d4-GCA	468.3	468.3	1.56	-125	-14	-3
d4-GCA*	468.3	74.0	1.56	-125	-64	-5

*: *Indicates ion pair for quantitation*

Table S2 Elution gradient for bile acids separation

Time (min)	flow rate (mL/min)	A (%)	B (%)	Curve
0.50	0.500	70.0	30.0	6
0.80	0.500	67.0	33.0	8
0.90	0.500	60.0	40.0	6
1.00	0.500	55.0	45.0	6
3.20	0.500	45.0	55.0	8
3.30	0.500	35.0	65.0	6
3.40	0.500	0.0	100.0	6
4.00	0.500	0.0	100.0	6
4.10	0.500	70.0	30.0	6
5.00	0.500	70.0	30.0	6

Table S3 Quantitative range and linearity for 15 bile acids

Bile acids	Quantifier transition	Calibration Range(nmol/L)	Linear Regression	R^2
CA	407.3/407.3	10~14000	y = 4.38511e-4 x + -7.78749e-4	0.99901
UDCA	391.3/391.3	3~5300	y = 7.10174e-4 x + 0.00295	0.99843
CDCA	391.3/391.3	6~8900	y = 5.68848e-4 x + -0.00127	0.99967
DCA	391.3/391.3	3~4400	y = 7.60477e-4 x + 0.00268	0.99909
LCA	375.3/375.3	5~6000	y = 0.01560 x + -0.05665	0.99659
GCA	464.3/74.0	4~7200	y = 0.00100 x + 0.01460	0.99946
GUDCA	448.3/74.0	2~9000	y = 0.00184 x + 0.00620	0.99792
GCDCA	448.3/74.0	3~6200	y = 0.00199 x + 0.00871	0.99977
GDCA	448.3/74.0	4~6700	y = 0.00174 x + -0.00104	0.99990
GLCA	432.3/74.0	3~4700	y = 0.00246 x + -0.00245	0.99953
TCA	514.3/80.0	5~6800	y = 5.67981e-4 x + -0.00189	0.99828
TUDCA	498.3/80.0	3~7900	y = 0.00240 x + 0.00341	0.99897
TCDCA	498.3/80.0	6~8700	y = 0.00105 x + -0.00263	0.99915
TDCA	498.3/80.0	7~8100	y = 0.00120 x + -0.00540	0.99874
TLCA	482.3/80.0	4~7200	y = 0.00221 x + -0.00111	0.99772

Table S4 The precision and average accuracy of 15 bile acids in LQC, MQC and HQC samples

	LQC		MQC		HQC	
**Bile Acids**	**Precision**	**Accuracy**	**Precision**	**Accuracy**	**Precision**	**Accuracy**
CA	9%	87%	5%	91%	4%	89%
CDCA	8%	103%	7%	102%	3%	105%
DCA	6%	108%	6%	107%	3%	105%
LCA	8%	85%	7%	86%	6%	86%
UDCA	6%	94%	3%	92%	2%	85%
GCA	7%	98%	5%	99%	3%	97%
GCDCA	8%	107%	7%	108%	5%	109%
GDCA	10%	106%	8%	113%	3%	112%
GLCA	9%	105%	7%	104%	5%	112%
GUDCA	9%	108%	5%	105%	4%	106%
TCA	6%	114%	5%	112%	4%	112%
TCDCA	8%	109%	5%	113%	3%	110%
TDCA	7%	108%	5%	110%	5%	110%
TLCA	8%	103%	7%	104%	6%	106%
TUDCA	6%	89%	4%	87%	2%	94%

Note: LQC, low quality control sample; MQC, medium quality control sample; HQC, high quality control sample

Supplementary Methods

To characterize patient-level treatment responsiveness while accounting for inter-individual heterogeneity in bile acid dynamics, a rule-based response framework was specified a priori. For each patient i and each bile acid j, treatment-associated changes were quantified as the difference between post-treatment and pre-treatment concentrations:

$$\Delta BA_{ij} = BA_{ij}^{\text{post}} - BA_{ij}^{\text{pre}},$$

where $BA_{ij}^{(t)}$ denotes the concentration of bile acid j in patient i at time point $t \in \{\text{pre}, \text{post}\}$.

Among all bile acids showing post-treatment decreases within each responder, the bile acid exhibiting the largest magnitude of decline was selected as the representative treatment-responsive feature. This bile acid was identified as:

$$j_i^* = \arg\min_j (\Delta BA_{ij}).$$

To construct a patient-level composite response index that captures the maximal treatment-associated change, a weighting function w_{ij} was defined such that:

$$w_{ij} = \begin{cases} 1, & \text{if } j = j_i^*, \\ 0, & \text{otherwise.} \end{cases}$$

This weighting function was determined once for each patient based on treatment-associated changes and was subsequently applied consistently to both pre-treatment and post-treatment bile acid profiles. Using this fixed weighting scheme, bile acid concentrations at each time point were aggregated into a single integrated bile acid value according to:

$$BA_i^{\text{agg}}(t) = \sum_{j=1}^{N} w_{ij} \cdot BA_{ij}^{(t)}, t \in \{\text{pre}, \text{post}\}.$$

A bile acid was considered to exhibit a treatment response if its concentration decreased after treatment ($\Delta BA_{ij} < 0$). To formalize this criterion, a binary indicator function was defined as follows:

$$I_{ij} = \begin{cases} 1, & \Delta BA_{ij} < 0, \\ 0, & \Delta BA_{ij} \geq 0. \end{cases}$$

At the patient level, treatment responsiveness was defined based on the presence of any bile acid decrease. Specifically, a patient i was classified as a responder if at least one bile acid satisfied the response criterion:

$$\sum_{j=1}^{N} I_{ij} \geq 1,$$

where N denotes the total number of bile acids analyzed.

A bile acid was considered responsive if it exhibited a post-treatment decrease. Patients were classified as responders if at least one bile acid showed a decrease following treatment, defined as:

$$Z_i = \begin{cases} 1, & \text{if } \sum_{j=1}^{N} I_{ij} \geq 1\,, \\ 0, & \text{otherwise.} \end{cases}$$

Response rate was defined as the proportion of patients classified as responders, calculated by dividing the number of responders by the total number of patients:

$$\text{Response Rate} = \frac{1}{M}\sum_{i=1}^{M} Z_i,$$

where M is the total number of patients.
